# Magnetic Resonance Imaging (MRI) Findings in a Case of Pigmented Villonodular Synovitis of the Knee

**DOI:** 10.7759/cureus.45806

**Published:** 2023-09-23

**Authors:** Iram Saifi, Shivali V Kashikar, Pratap Parihar, Azeem I Saifi, Khizer K Ansari

**Affiliations:** 1 Radiodiagnosis, Jawaharlal Nehru Medical College, Datta Meghe Institute of Higher Education and Research, Wardha, IND; 2 Medicine and Surgery, Jawaharlal Nehru Medical College, Datta Meghe Institute of Higher Education and Research, Wardha, IND

**Keywords:** localised pvns, diffuse pvns, mri of pvns, pvns, tenosynovial giant cell tumour

## Abstract

Pigmented villonodular synovitis (PVNS) is a rare benign condition of tenosynovial proliferation that mostly affects the knee joint. In this case report, we present a 39-year-old female with a ten-year history of gradual progression in the size of painful soft tissue swelling in her left knee. Our case report emphasizes the MRI's ability to provide detailed information on tendon sheath and synovium involvement, as well as extensive extra-articular involvement and hemosiderin deposition.

## Introduction

Pigmented villonodular synovitis (PVNS) represents a benign proliferative anomaly of the synovial lining, primarily observed in joints, with a notable predilection for the knee joint. This condition warrants clinical attention due to its potential to cause joint pain and limited mobility, but there is a significant risk of recurrence, necessitating a comprehensive evaluation and tailored management strategies. It has monoarticular and polyarticular involvement, although it is rare. Clinical features include soft tissue swelling, which can be painless in localized form and even become painful in diffuse form. Swelling is usually gradually progressive for many years causing a limited range of movements [1].

## Case presentation

A 39-year-old female complained of soft tissue swelling in her left knee for 10 years, which was painful and gradually progressive in size with decreased movement. There was no history of trauma, surgery, diabetes mellitus, or hypertension. On local examination, there is non-tender large diffuse swelling, soft in consistency with no elevated temperature noted in the anterior and posterior aspects of the left knee joint. The systemic examination was within normal limits. The patient was advised of magnetic resonance imaging (MRI) of the left knee joint.

**Figure 1:**
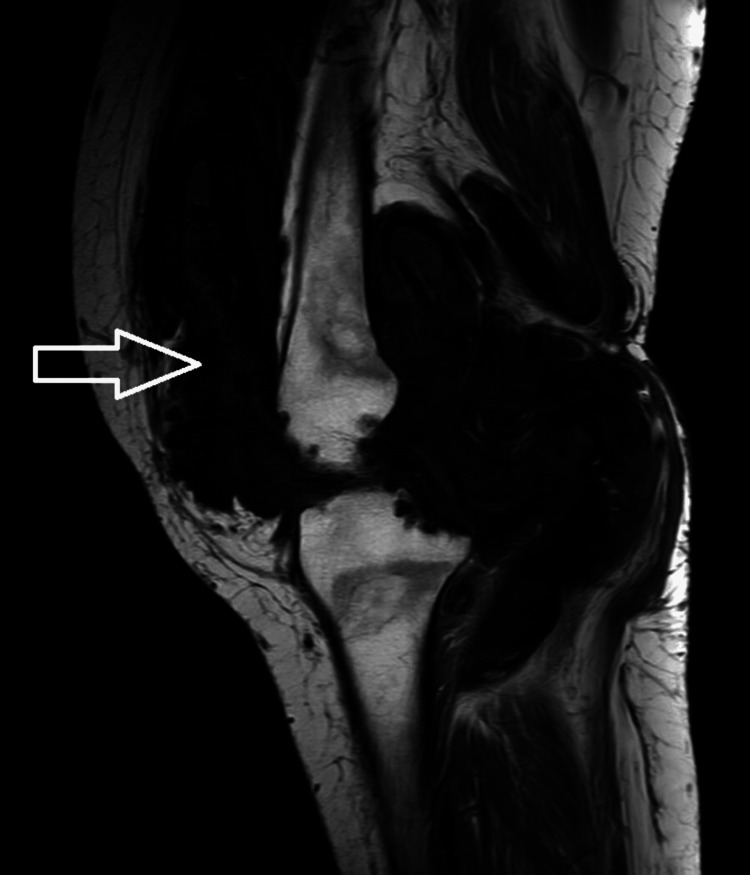
Sagittal T1WI showing diffuse irregular thickening and proliferation of the synovial lining of anterior and posterior aspects of the knee joint, suprapatellar bursa, deep infrapatellar bursa, popliteal, semimembranosus bursa, Hoffa's fat pad, forming nodular and villous projections appearing hypointense (white arrow) on T1.

**Figure 2:**
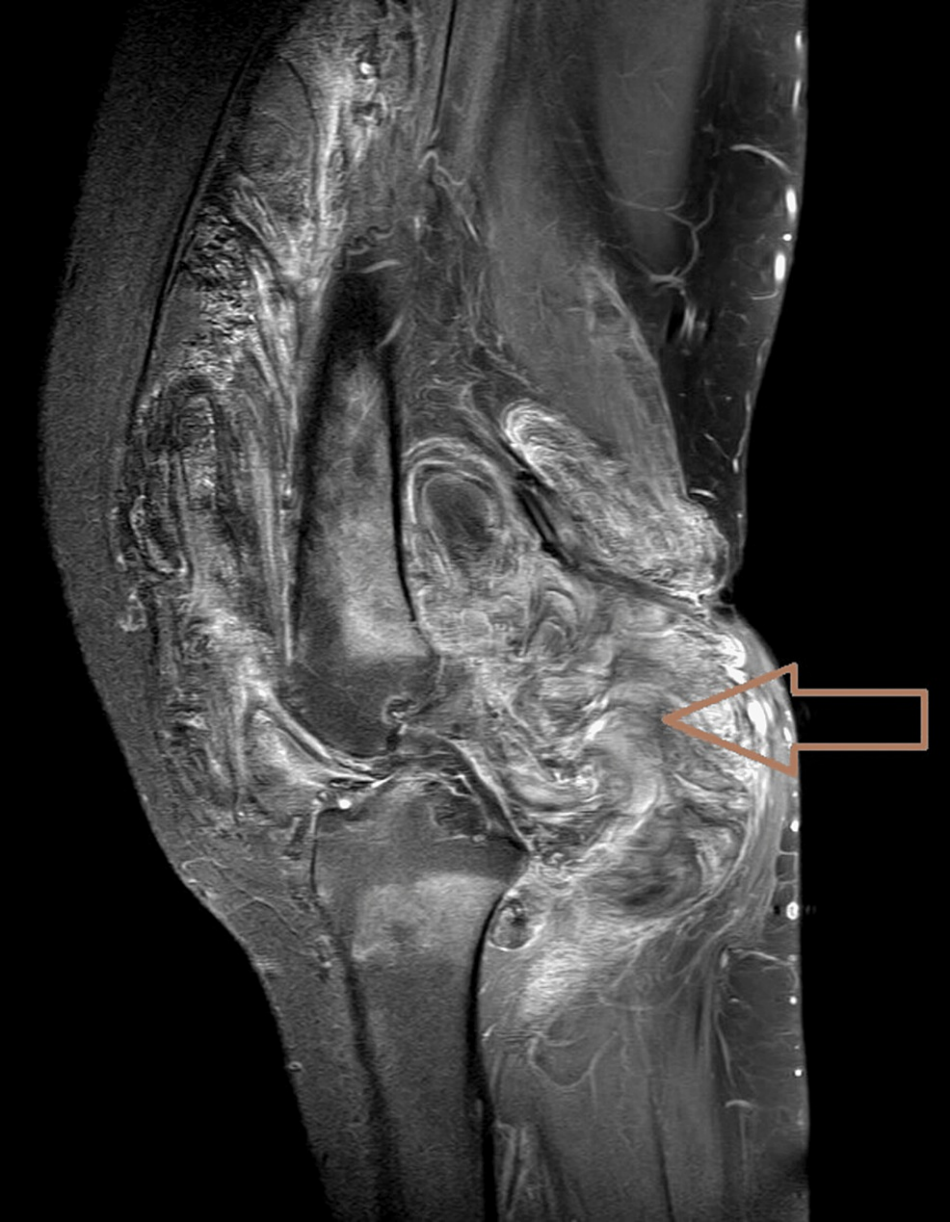
Sagittal T1W contrast image showing enhancing (brown arrow) diffuse irregular thickening and proliferation of the synovial lining of the anterior and posterior aspects of the knee joint.

**Figure 3:**
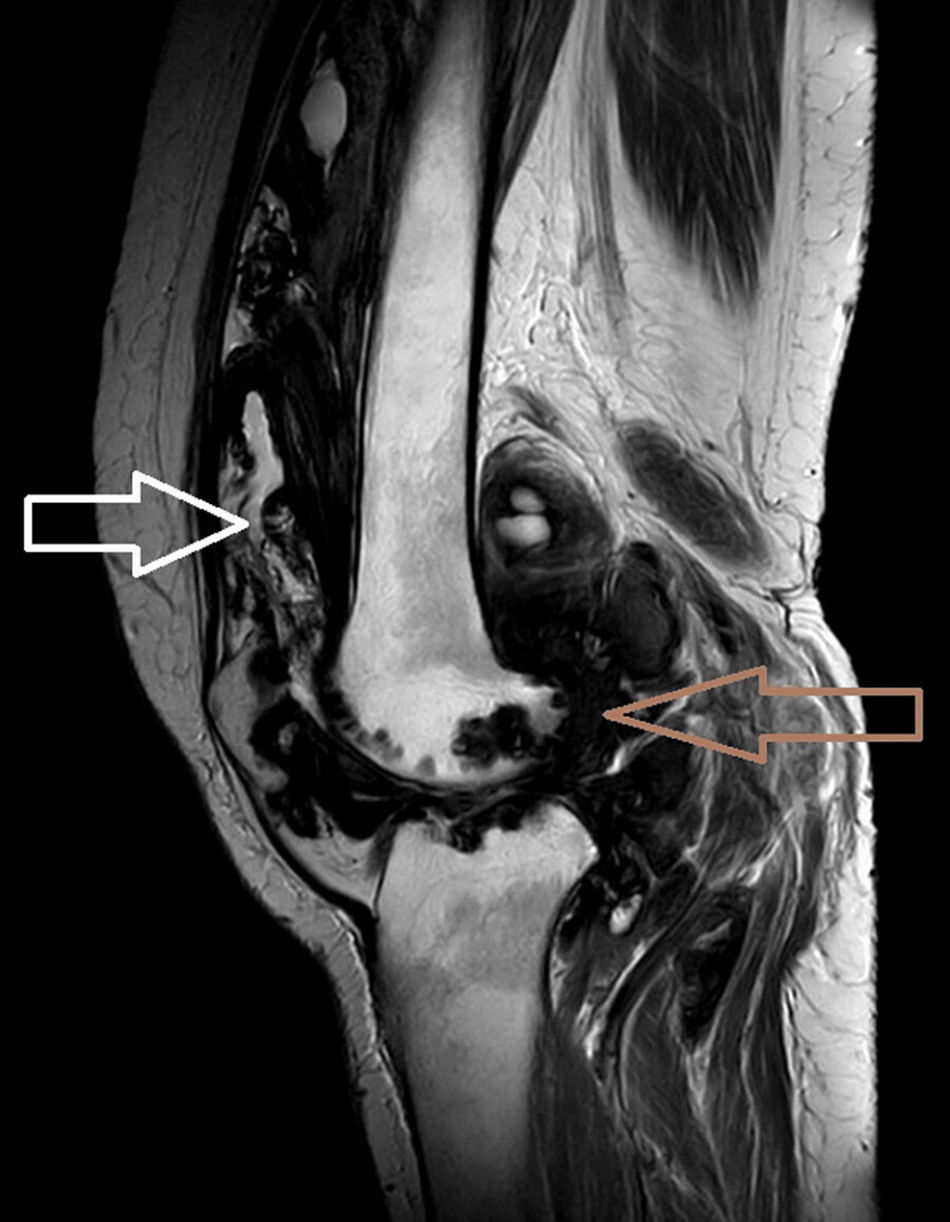
Sagittal T2WI showing diffuse irregular thickening and proliferation of the synovial lining of anterior and posterior aspects of the knee joint, suprapatellar bursa, deep infrapatellar bursa, popliteal, semimembranosus bursa, Hoffa's fat pad, forming nodular and villous projections showing intermediate signal on T2 with a few areas appearing hyperintense due to joint effusion.

**Figure 4:**
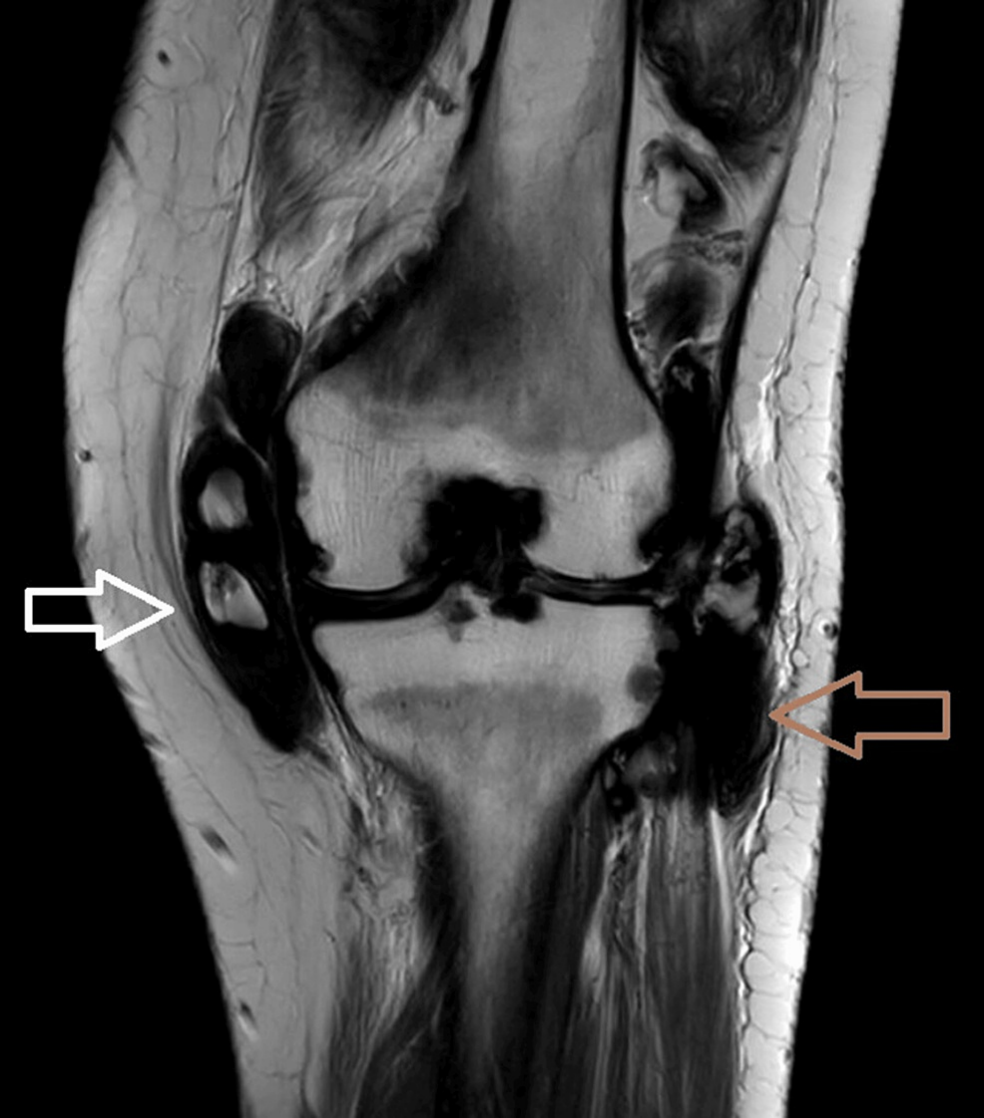
Coronal T2WI showing diffuse irregular thickening and proliferation of the synovial lining of the medial (white arrow) and lateral compartments (brown arrow) forming nodular and villous projections.

**Figure 5:**
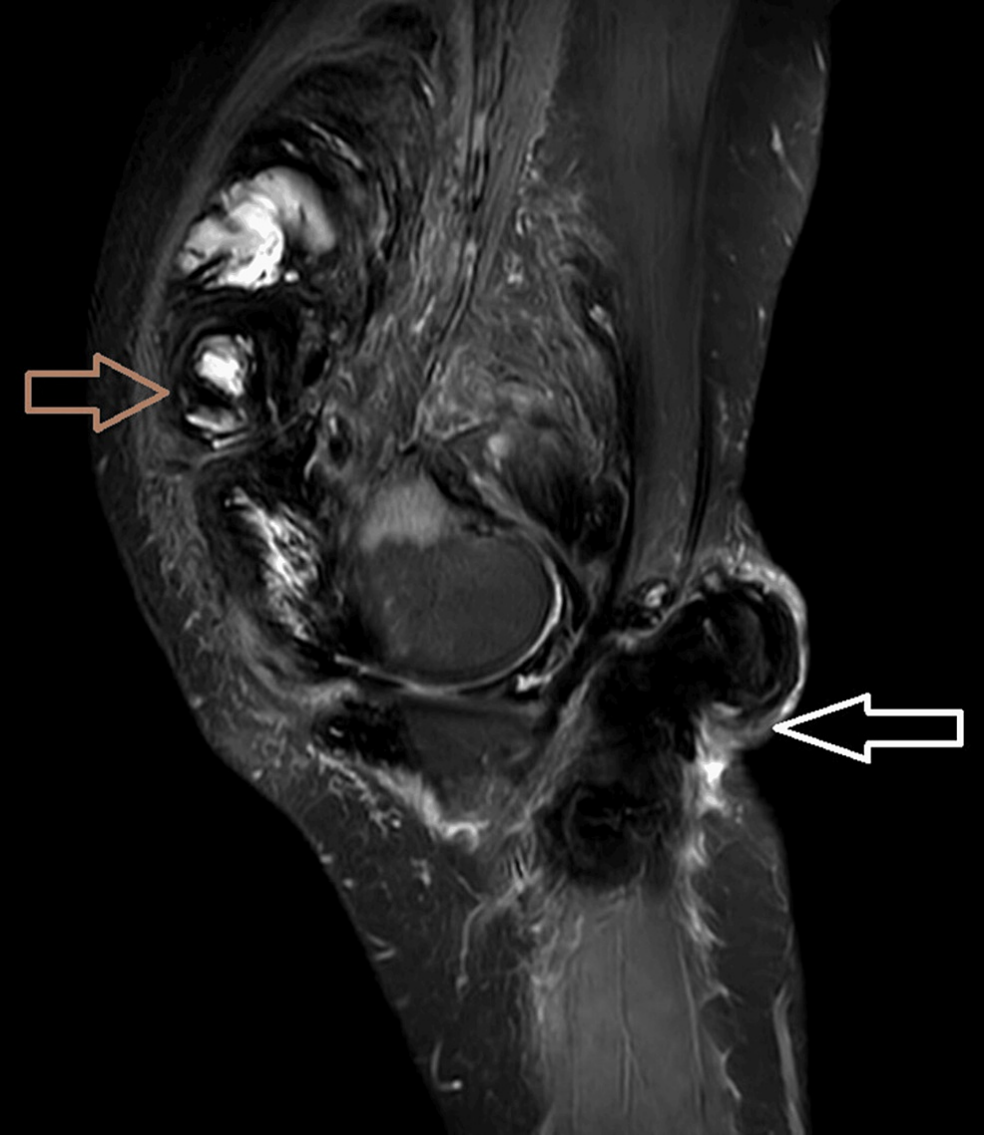
Sagittal PDW image showing diffuse irregular thickening and proliferation of the synovial lining of the anterior (brown arrow) and posterior aspects of the knee joint (white arrow) appearing predominantly hyperintense. PDW: proton density-weighted imaging.

MR imaging showed variably enhancing diffuse irregular thickening and proliferation of the synovial lining of the anterior and posterior aspects of the knee joint (Figures 1-3). This condition also affected the medial and lateral compartments of the knee (Figure 4), as well as the supra-patellar bursa, deep infra-patellar bursa, popliteal bursa, semi-membranosus bursa, and Hoffa's fat pad. These abnormalities appeared as nodular and villous projections that were hypointense on T1 imaging (Figure 1), had an intermediate signal on T2 imaging (Figure 3), and were predominantly hyperintense on proton density fat saturation sequence (PDFATSAT) (Figure 5).

**Figure 6:**
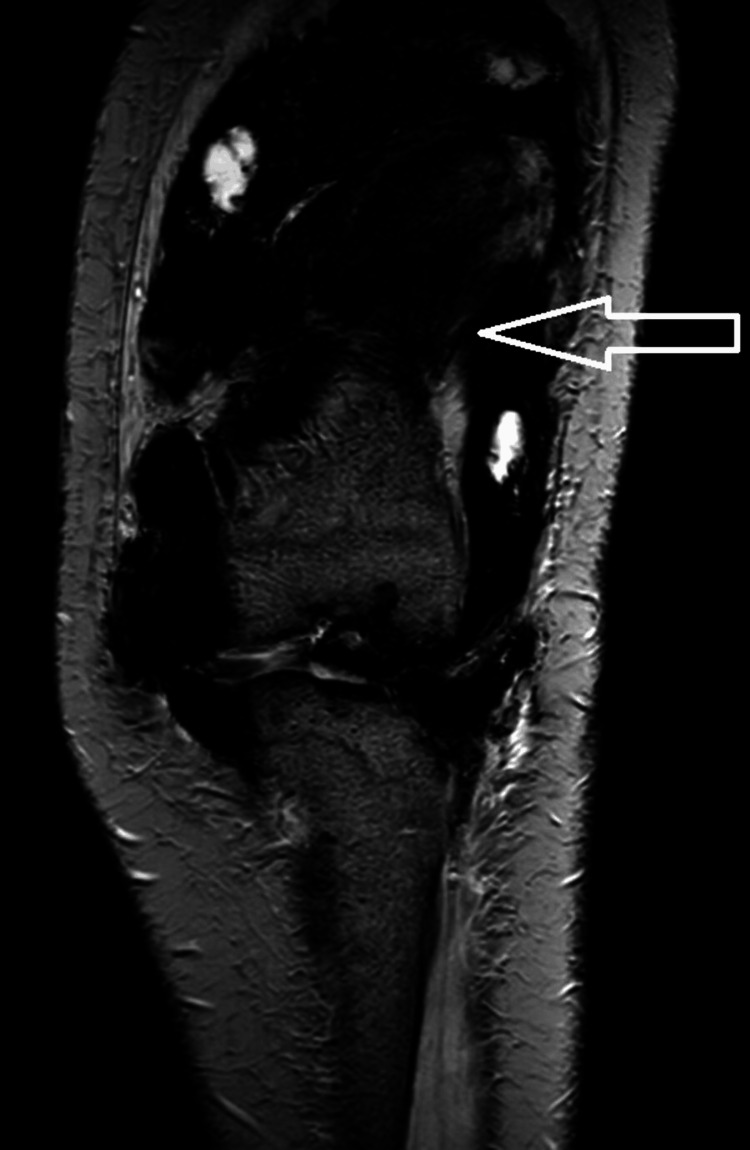
Coronal GRE imaging showing blooming (white arrow) in villous and nodular projections suggestive of hemosiderin deposition. GRE: gradient echo.

**Figure 7:**
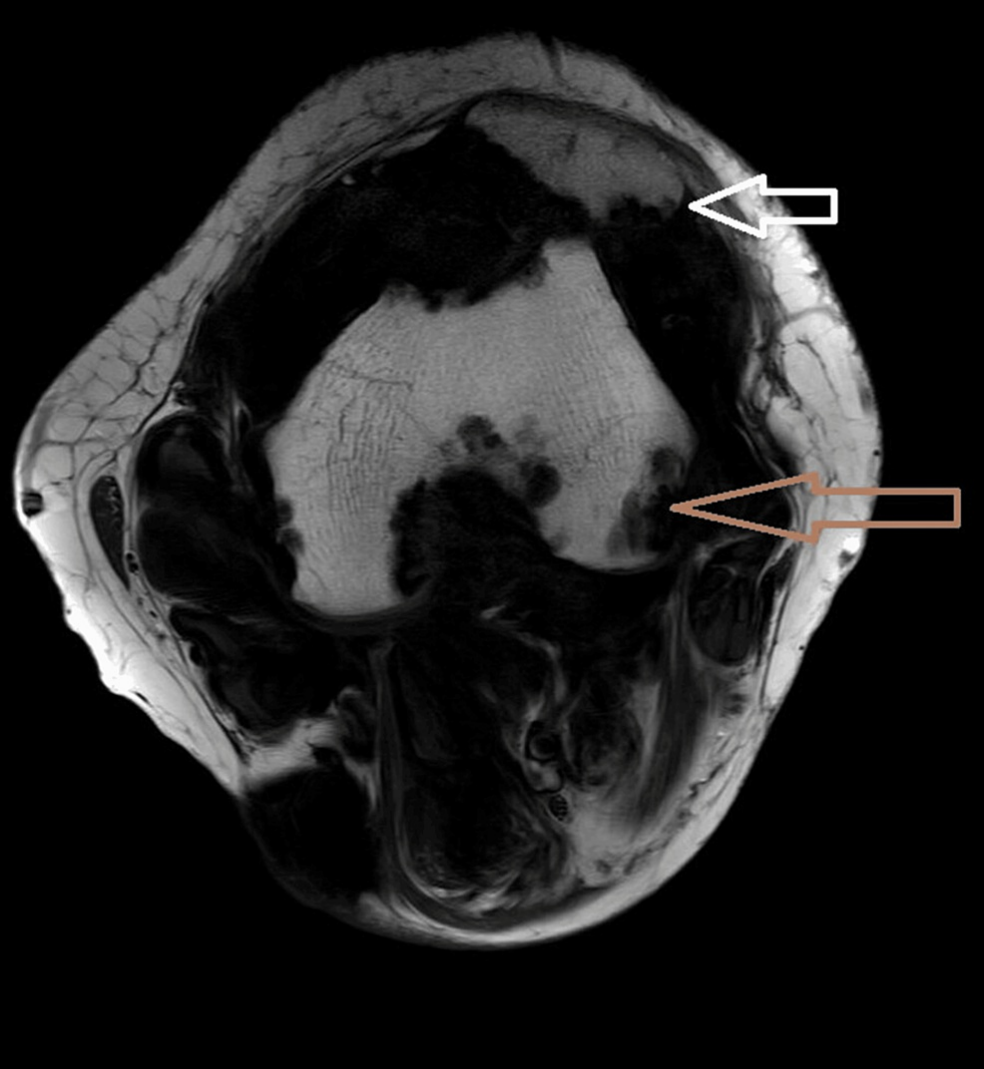
Axial T1WI showing synovial masses imprinting the surface of bones and causing pressure erosions of the patella (white arrow) and femur (brown arrow).

The synovial membrane and some nodular projections exhibit low T1/T2 signal intensity and blooming on gradient echo (GRE) (Figure 6), suggesting hemosiderin deposition. ﻿﻿Altered signal intensity lesions were observed in the distal femoral condyle, proximal tibia, fibula, muscles, ligaments, menisci, and subcutaneous plane on the posterior aspect of the knee joint. These lesions appeared hypointense on T1, T2, and PDFATSAT sequences and demonstrated blooming on GRE, indicating intraosseous, muscular, ligamentous, and subcutaneous extension. ﻿Synovial masses imprint the surface of the adjacent bone, causing erosion of the patella, distal femur, proximal tibial condyle, and fibula (Figure 7). ﻿﻿Moderate joint effusion noted. The patient was advised for extensive surgical treatment and postoperative radiotherapy in our case since the chances of recurrence are very high due to the extensive involvement of the disease. 

## Discussion

Pigmented villonodular synovitis (PVNS) is recognized as a tenosynovial giant cell tumor, characterized by its complex histopathological nature. This condition exemplifies the intriguing interplay between synovial tissues and giant cell proliferation, making it a subject of considerable interest for clinicians and researchers alike. Its variable clinical presentation and potential for local joint destruction underscore the importance of comprehensive diagnostic evaluation and the development of targeted therapeutic strategies. This condition is mostly benign and affects synovium and tendons. It can affect intra-articularly as well as extra-articular involvement is also known. PVNS usually involves one joint, but sometimes multiple joint involvement is also seen. They are present equally in males and females. The mean age of presentation is 30-40 years. It is divided into localized and diffuse types [1-3].

Localized type of PVNS occurs usually in the finger around the tendon sheath. Macroscopically, they look like nodular soft tissue swelling [4]. Diffuse type of PVNS involves intra-articular and extra-articular compartments. They occur most commonly in large joints such as the knee. However, hip, ankle, shoulder, and elbow joints can also be involved. Diffuse types of PVNS have more female predilection [1]. PVNS appears as soft tissue swelling on the x-ray. It can present as bony erosion at a later stage. On computed tomography, it shows joint effusion and thickened synovium appearing as hyperdense soft tissue swelling due to hemosiderin accumulation. In the later stage of the disease, bony erosion is well appreciated on CT. Ultrasonography provides good detail in the localized types of PVNS and also shows the communication of swelling within the tendon sheath in fingers. It appears as a homogenously hypoechoic lesion with little internal vascularity on Doppler [5].

MRI is considered the most important investigation to see intra-articular and extra-articular involvement in the case of PVNS. It shows the villous and nodular projection of the synovium with hemosiderin accumulation. It appears hypointense on T1 and T2, with few areas of hyperintensity on T2 due to fluid accumulation. It appears hyperintense on PDFATSAT and short tau inversion recovery (STIR) sequences and shows blooming on GRE due to the presence of hemosiderin [2]. In contrast administration, it appears variably enhancing in the diffuse dorm and shows moderate enhancement in the localized form [6]. PVNS not only affects synovium, but in extensive disease, it can involve tendons, ligaments, muscles, and even bone involvement in pressure erosions [7]. Treatment includes surgical excision in localized form. In diffuse form and complete synovectomy, extensive surgery is required along with radiotherapy. Follow-up is extremely important since the recurrence rate is higher in diffuse PVNS, which is nearly 30-35% [1]. 

## Conclusions

This case report highlights the importance of MRI in diagnosing any knee swelling, which can vary from normal joint effusion to extensive tumor-like conditions that can be benign or malignant. It also tells us about the involvement of soft tissue structure around the joint, extra-articular involvement, and some bony involvement details. MRI helps categorize the disease in diffuse and localized forms and delineates the involvement of different structures around the joint. It helps in surgical planning and also helps in assessing the need for radiotherapy since the recurrence of this condition is higher. 
